# Amyotrophic lateral sclerosis in seven provinces of Chinese mainland: A cross-sectional survey from 2015 to 2016

**DOI:** 10.3389/fnagi.2022.946353

**Published:** 2022-09-15

**Authors:** Jie Zhang, Xiaoqing Liu, Huiting Liang, Shengyuan Xu, Xiaohua Wang, Renshi Xu

**Affiliations:** ^1^Department of Neurology, First Affiliated Hospital of Nanchang University, Nanchang, China; ^2^Jiangxi Province Center for Disease Control and Prevention, Nanchang, China; ^3^Department of Neurology, Jiangxi Provincial People's Hospital, Clinical College of Nanchang College, First Affiliated Hospital of Nanchang Medical College, Nanchang, China; ^4^Department of Geriatrics and General Practice/General Family Medicine, Jiangxi Provincial People's Hospital, Clinical College of Nanchang College, First Affiliated Hospital of Nanchang Medical College, Nanchang, China

**Keywords:** epidemiology, amyotrophic lateral sclerosis, Chinese mainland, population, hospital

## Abstract

**Background:**

The large-scale survey about amyotrophic lateral sclerosis (ALS) based on both population and hospitals in the Chinese mainland has been deficient at present. To this end, we conducted a cross-sectional survey about ALS based on the population and hospitals in seven provinces of the Chinese mainland in 2015–2016.

**Methods:**

We surveyed patients with ALS in seven provinces in eastern, middle, and western China. Among them, 13 prefecture-level cities, 13 municipal districts, 13 counties, 26 streets, 52 communities, 39 towns, and 78 administrative villages were selected for the population-based survey. Totally, 13 class-3 general hospitals, 13 class-2 general hospitals, and 26 street health centers or community health service centers in urban districts, and 13 county-level general hospitals, 39 township health centers, and 78 village clinics in rural districts were recruited for the hospital-based survey.

**Results:**

Among the Chinese mainland, the total prevalence of ALS was slightly lower than that of the world's other nations or districts. The male patients were more than female patients. The prevalence of rural residents was more than that of urban residents. The prevalence of farmers was higher than that of other professions. The majority of ALS was not accompanied by other chronic diseases. The peak onset age of ALS was higher, familial ALS (fALS) cases were slightly more, and the average survival duration of sporadic ALS (sALS) was slightly lower compared with the previous investigation data. The hospitalization expenses of almost 60% of ALS were not more than 10,000 Chinese Yuan.

**Conclusion:**

Hospitalization expenses in our survey objectives were the lowest in the current reported countries and districts. A farmer was a possible higher risk profession for ALS, the majority of ALS were not accompanied by other chronic diseases. Our survey provided more information on the epidemiology of ALS worldwide and supplied the deficiency of epidemiology survey about ALS from the Chinese mainland.

## Highlights

- ALS epidemiological characteristics are in seven provinces of the Chinese mainland in 2015–2016.
- This study is the currently largest and most comprehensive survey about ALS on Chinese mainland.
- Farmer is a possible higher risk profession of ALS in the Chinese mainland.
- This survey is an important addition to both population-based and hospital epidemiology.

## Introduction

Amyotrophic lateral sclerosis (ALS), also known as Lou Gehrig's disease in America, is a disaster disease, which is characterized by the selective and progressive death of the motor neurons in the cerebrum, brain stem, and spinal cord (Zarei et al., 2015). In some countries such as the United Kingdom, India, and Australia, the term motor neuron disease (MND) is commonly used to replace ALS (Zarei et al., 2015), while in other countries including America and China often use the MND term to call for a disease group of four subtypes including ALS, medullae (bulbar) palsy, progressive spinal muscular atrophy, and primary lateral sclerosis, and among them, ALS is the most common subtype (Ludolph et al., 2012). The early symptoms of ALS are mild and easy to be confused with other diseases. Patients may just feel weakness, muscle subsultus (beat or jump), fatigue at the initial stage, and gradually develop the atrophy of limbs, laryngeal, and pharynx muscles, and subsequently expand to the atrophy of whole body muscle, which results in the motor dysfunction of limbs, the difficulty of speech and swallow, and eventually generate the respiratory failure. Most patients with ALS die from respiratory failure (Zarei et al., 2015). The etiology and pathogenesis of ALS are not known in 90% to 95% of the patients with ALS. Only about 5% to 10% of ALS cases are inherited (Zarei et al., 2015).

The current diagnosis of ALS is mainly based on the patient's signs and symptoms, and supplemented with some related auxiliary examinations to rule out other mimic diseases (Silani et al., 2011; Statland et al., 2015). No useful cure for ALS is found at present. Based on the information from current epidemiological investigations, ALS affects slightly more men than women, the age distribution of ALS peaks at 55 to 60 years for both men and women worldwide, and the onset age usually is around 60 years (Kiernan et al., 2011). The average survival periods from onset to death usually are 3–5 years. About 10% of patients with ALS survive longer than 10 years (Salameh et al., 2015).

In many countries and districts of the world, the epidemiological data of ALS has been unknown yet (Kiernan et al., 2011). In Europe, the prevalence of ALS is about 2.2 people per 100,000 per year for all ages (Kiernan et al., 2011). In the United States, the prevalence of ALS is ~1.5 people per 100,000 per year for all ages (Mehta et al., 2016). The epidemiological survey of ALS in the Chinese mainland has been very deficient up to now. To obtain more estimation on the world epidemiology features of ALS, the epidemiological survey of ALS worldwide is needed to be improved further. To this end, we conducted a cross-sectional survey based on both population and hospitals in seven provinces of the Chinese mainland from 2015 to 2016, including prevalence, sexuality, onset age, geographical location, profession, chronic disease history, familial history, survival time, and both diagnosis and cure condition (hospitalization expenses). The detailed and large epidemiologic characteristics survey of ALS has been very deficient in the Chinese mainland. This epidemiologic investigation is the largest and most comprehensive survey about ALS on the Chinese mainland. Our study is an important addition to the population and hospital-based epidemiological survey of ALS in the Chinese mainland. Our survey supplied clinical data about the prevalence, the male and female ratio, the onset age, the rural and urban residents' onset, profession, the ratio of familial ALS, the major accompanying chronic diseases, the average survival duration, and the hospitalization expense of ALS in the population of the Chinese mainland, and more detailed epidemiologic information of ALS, and provided the epidemiologic evidence for the clinical prevention and treatment of ALS. Our data supplied the deficiency of ALS epidemiology in the Chinese mainland and provided more information on the world epidemiology of ALS.

## Methods

### Study design and procedures

**Figure 1:**
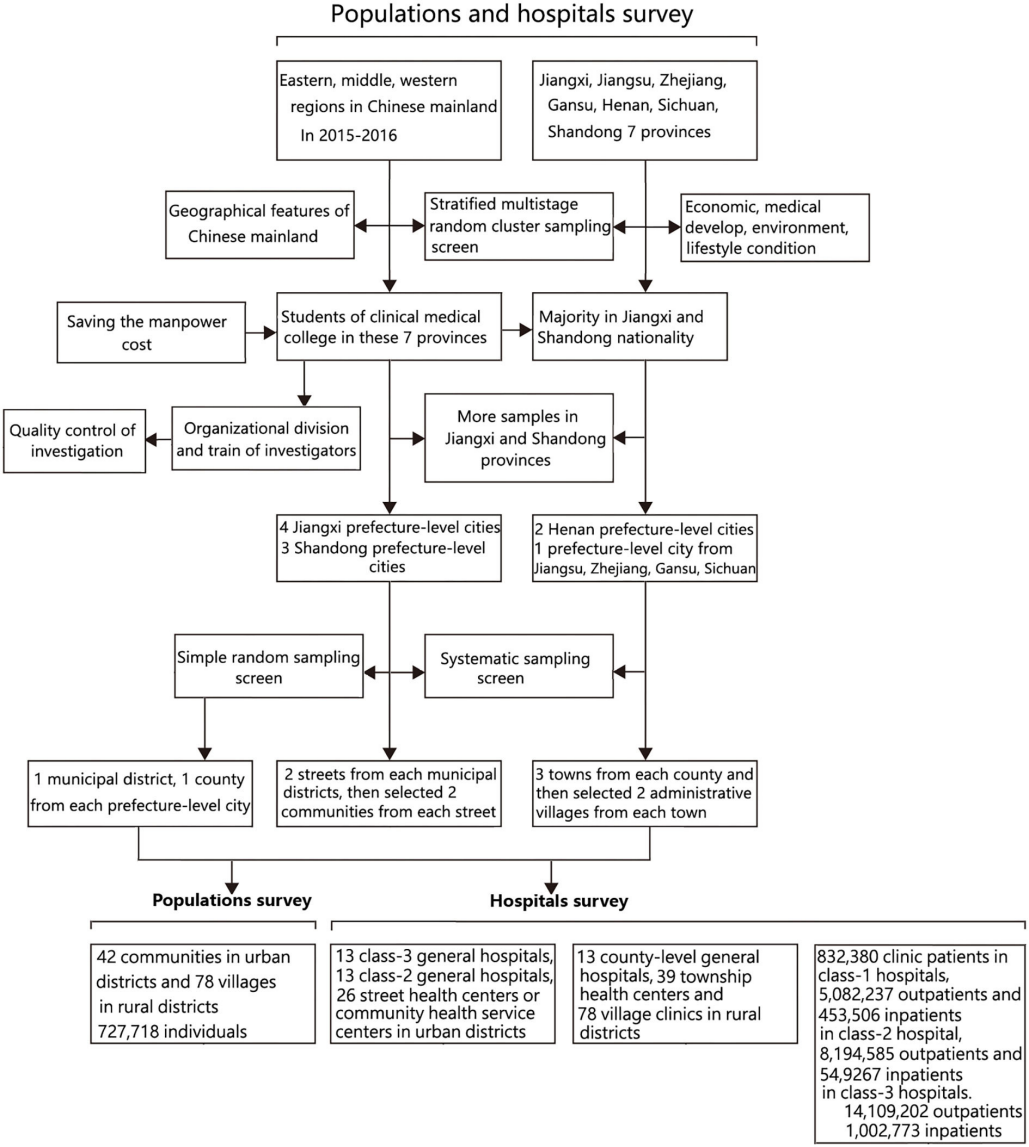
Selection of participants for population and hospital survey.

In 2015–2016, we undertook a nationwide cross-sectional survey based on population and hospitals jointing Jiangxi Province Center for Disease Control and Prevention to more comprehensively understand the epidemiological characteristics of ALS in the Chinese mainland (Figure 1). This survey was approved by the ethics committee of the First Affiliated Hospital of Nanchang University (approval number: 2014-06-01). We obtained multilevel approvals from the local Center for Disease Control and Prevention, selected hospitals, and selected communities and villages. All participants were to participate voluntarily and gave oral consent.

### Screening of investigation regions

Based on the classification of the eastern, middle, and western regions of our country, we used a stratified multistage random cluster sampling design to obtain representative samples of people in general populations and hospitals at the different levels of districts. We selected seven provinces including Jiangxi, Jiangsu, Zhejiang, Gansu, Henan, Sichuan, and Shandong province. These districts differed in the economic, medical developmental, environmental, and lifestyle conditions, which possessed the representation statistically.

**Figure 2:**
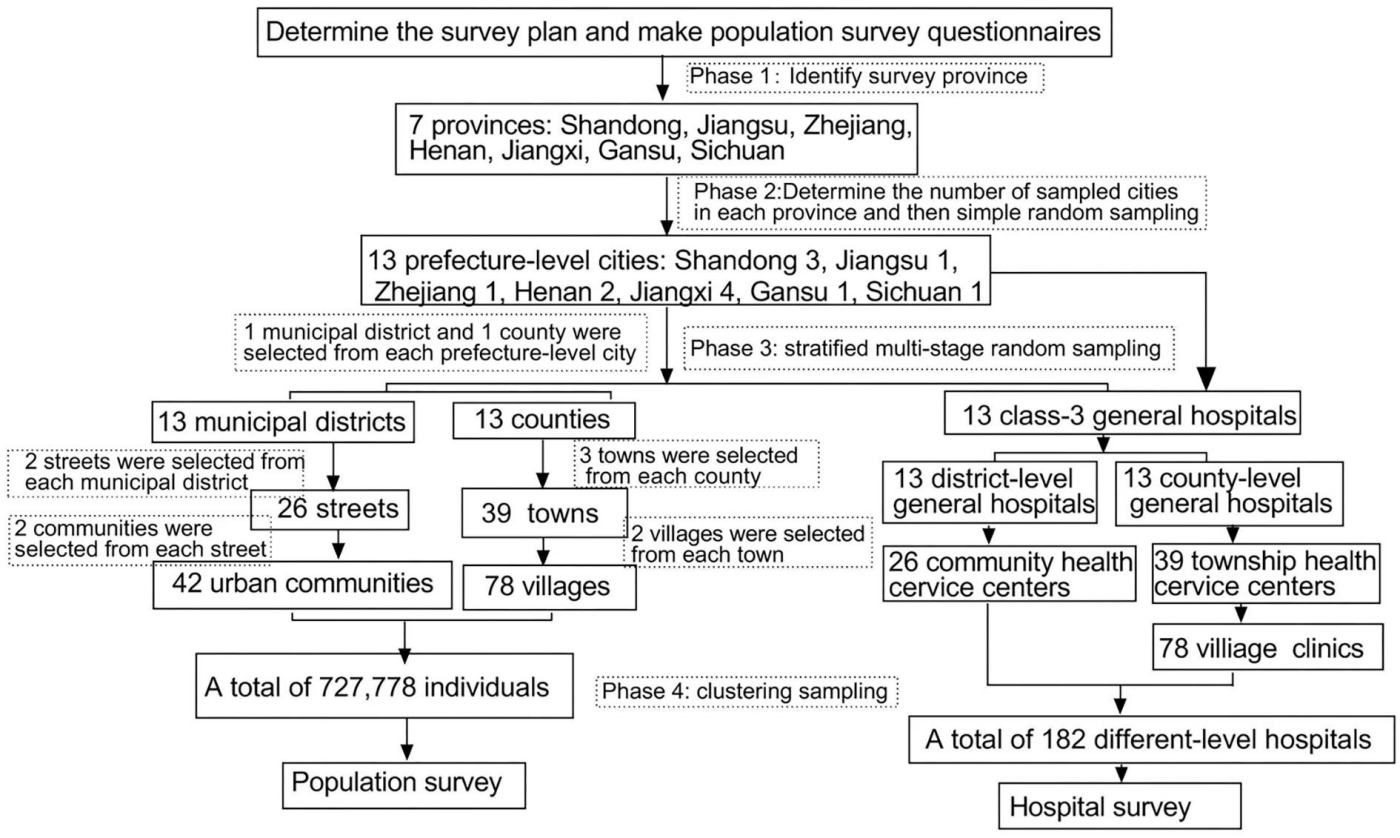
Flow chart of epidemiological survey.

To save manpower cost, these students whose household registers were in these seven provinces and who studied in the Clinical Medical College of Nanchang University were recruited to perform this survey. Because the students of Jiangxi and Shandong nationality occupied the majority of them in the University of Nanchang at that time, we selected more samples from these two provinces than from other provinces. By probability proportionate to size sampling, we sampled four prefecture-level cities from Jiangxi province, three prefecture-level cities from Shandong province, two prefecture-level cities from Henan province, and one prefecture-level city for each province from the other four provinces (Jiangsu, Zhejiang, Gansu, and Sichuan). We surveyed 13 prefecture-level cities in total. By simple random sampling method, we selected one municipal district and one county from each prefecture-level city. We selected two streets from each municipal district and selected two communities from each street using the systematic sampling method. We selected three towns from each county and then selected two administrative villages from each town by the systematic sampling method. All regions selected based on the above-described statistically designed method were recruited as the investigation districts in this survey. The detail about the screening of investigation regions was in Figure 2.

### Organizational and division of investigators

Dr. Jie Zhang took the general directors of this survey, who were responsible for the establishment of schemes, personnel training, overall analysis of data, and report writing. About three to four neurologists of hospitals in prefecture-level cities of each province acted as the regional supervisors, and were responsible for organizing and implementing the schemes of prefecture-level cities, on-site supervision and the quality control, analysis of data, and report writing of prefecture-level cities. We trained 23 medical students including undergraduate and postgraduate students as investigators, whose household registers were in the selected prefecture-level cities. They were responsible for the implementation of an on-site investigation plan, the collection, arrangement, and analysis of original data, and report writing of municipal districts and counties.

### Population survey schemes

For each surveyed prefecture-level city, we extracted four communities and six villages. We finally selected 42 communities in urban districts and 78 villages in rural districts totally. We produced questionnaires, and took a household as a unit to conduct home visits, excluding those family members who had died or lived <3 months every year in the surveyed district (Figure 2).

### Hospital survey schemes

For the surveyed municipal districts (two streets) and sub-districts under its jurisdiction (four communities) of each prefecture-level city in this hospital survey, we, respectively, extracted a class-3 general hospital, a class-2 general hospital, and two street health centers or community health service centers. For the surveyed counties (three towns) and sub-districts under its jurisdiction (six administrative villages) of each prefecture-level city in this hospital survey, we, respectively, extracted a county-level general hospital, three township health centers, and six village clinics. We finally selected 13 class-3 general hospitals, 13 class-2 general hospitals, and 26 street health centers or community health service centers in urban districts, and 13 county-level general hospitals, 39 township health centers, and 78 village clinics in rural districts totally. All hospitals selected based on the above-described statistically designed method were recruited as investigation subjects in this hospital survey (Figure 2).

### Diagnosis of patients with ALS

The diagnosis of ALS followed the standardized diagnostic procedures of ALS (Brooks et al., 2000; Ludolph et al., 2015; Johnsen et al., 2019). In the population survey, investigators briefly asked questions about demographics and medical history at initial household assessments. If there were suspicious patients with ALS, they would be reported to the regional supervisors and taken to the local class-3 general hospital for a further diagnosis. To improve the accuracy of preliminary diagnoses, we repeated neurological examinations in all confirmed and suspected cases after 3 and 6 months using electromyography and the related lab examination by assessing disease progression, and re-examinations excluded those patients with ALS who refused another assessment, had died, or were lost to follow-up in the local class-3 general hospital. The neurologist director Prof. Renshi Xu made the final diagnosis based on all available information provided by investigators.

In the hospital survey, we searched patients with ALS in inpatients and outpatients through databases of hospitals. Once suspicious patients were found, they would be reported to regional supervisors, and if the patient had never been diagnosed in a tertiary hospital and could be contacted, he or she would be taken to the local class-3 general hospital for further diagnosis. To improve the accuracy of ALS diagnoses, we repeated neurological examinations in all definite and suspected cases following-up to 3 to 6 months using electromyography and related laboratory tests to further assess the ALS diagnosis in the local class-3 general hospital. Multiple investigations were conducted to exclude those patients who refused the further assessment, had died, or lost the follow-up. The neurologist director Prof. Renshi Xu made the final diagnosis based on all available information provided by investigators.

For all definite diagnostic ALS cases, we produced the investigation of questionnaires that involved general health situations, symptoms and signs, demographics, medical histories, profession, treatment processes, and costs.

### Quality control of investigation

Before the formal investigation, we selected Nanchang city to carry out a preliminary test to further improve the investigation plan and train all investigators (23 medical students) for 3 weeks in Nanchang city. Before finishing the household or hospital survey, investigators would conduct a comprehensive check of questionnaires. If there was any doubt, it was necessary to re-check and verify investigation contents, correct mistakes, and fill in missing items in time. The completion rate of the questionnaire investigation should be ≥99% and the error rate should be <1%. Regional supervisors should complete unqualified questionnaires and contact investigators to ascertain facts in time. During the investigation of fieldwork, staff members of the local Center for Disease Control and Prevention helped to communicate with communities or hospitals and supervisors monitored the on-site investigation interview of investigators.

### Statistical analysis

We used the SPSS statistical software of version 20.0 (SPSS, Chicago, IL, USA) for both data collection and management. All data of the cross-sectional survey based on population and hospitals including the epidemiological characteristics data of ALS were performed the statistical analysis by the SPSS software of version 20.0.

## Results

### Population survey

Our population survey covered 727,718 individuals, consisting of 160,983 (22.12%) Jiangxi province residents, 14,600 (2%) Jiangsu province residents, 10,600 (1.46%) Zhejiang province residents, 20,786 (2.86%) Gansu province residents, 52,081 (7.16%) Henan province residents, 18,950 (2.60%) Sichuan province residents, and 449,718 (61.80%) Shandong province residents (Table 1). The prevalence of ALS in seven provinces of the Chinese mainland was 1.24/100,000. Of these provinces, the prevalence of ALS in Zhejiang province was the highest (9.43/100,000) and the prevalence of ALS in Jiangsu province, Gansu province, Sichuan province, and Shandong province were 0/100,000 (Table 1).

**Table 1:** Prevalence of ALS in 7 provinces of China mainland in population survey.

**Districts**	**Number of ALS patients/ respondents**	**Number of respondents / total respondents (%)**	**Prevalence of ALS (1/100,000)**
Jiangxi Province	6**/**160,983	22.12	3.73
Jiangsu Province	0**/**14,600	2.00	0.00
Zhejiang Province	1**/**10,600	1.46	9.43
Gansu Province	0**/**20,786	2.86	0.00
Henan Province	2**/**52,081	7.16	3.84
Sichuan Province	0**/**18,950	2.60	0.00
Shandong Province	0**/**449,718	61.80	0.00
Total	9**/**727,718		1.24

Our population survey consisted of 478,402 (65.74%) urban residents and 249,316 (34.26%) rural residents. The prevalence of ALS in rural residents (2.01/100,000) was higher than that in urban residents (0.84/100,000) (Table 2). Of the nine patients with ALS with a final diagnosis, seven (77.78%) individuals were male. The average onset time of sporadic ALS (sALS) was 54.57±10.65 years of age, the peak onset time was around 50 years old. In that, seven (77.78%) individuals were farmers and two (22.22%) had a chronic disease or ALS family history. The survival duration of sALS was 2.07±1.08 years, and familial ALS (fALS) ranged from 6 to 8 years. Among them, the ALS prevalences in male patients and farmers were in the higher percentage (Table 3).

**Table 2:** Prevalence of ALS in urban and rural districts of China mainland in population survey.

**Districts**	**Number of ALS patients/** **respondents**	**Respondents/** **total respondents (%)**	**Prevalence of ALS (1/100,000)**
Urban districts	4**/**478,402	65.74	0.84
Rural districts	5**/**249,316	34.26	2.01
Total	9**/**727,718		1.24

**Table 3:** General clinical and demographic information of ALS patients identified in population survey.

**ID**	**Gender**	**Age**	**Profession**	**Medical history**	**Family history**	**Survival duration**
1	Male	65	Farmer	Unknown	None	3 years
2	Male	40	Farmer	Unknown	None	1 years
3	Male	45	Truck driver	Unknown	None	0.5 years
4	Male	62	Farmer	Unknown	None	1 years
5	Female	74	Farmer	Unknown	None	4 years
6	Female	46	Unemployed	Diabetes	None	3 years
7	Male	31	Farmer	Hepatitis	ALS	8 years
8	Male	40	Farmer	Unknown	ALS	6 years
9	Male	50	Farmer	Unknown	None	2 years

### Hospital survey

All surveyed class-1 hospitals in the hospital survey involved 26 street health centers or community health service centers in urban districts, 39 township health centers, and 78 village clinics in rural districts. There were only outpatient departments in these hospitals. Two individuals with ALS were identified among 832,380 outpatients in these hospitals. The prevalence of ALS in class-1 hospital outpatients was 0.24/100,000 (Table 4).

**Table 4:** Ratio of ALS outpatients and inpatients in different level hospitals of 7 provinces of China mainland in 2015–2016.

	**Number of ALS outpatients/ total outpatients (1/100,000)**	**Number of ALS inpatients/ total inpatients (1/100,000)**
Class-1 hospitals	2**/**832,380 (0.24**/**100,000)	
Class-2 hospitals	12**/**5,082,237 (0.24/100,000)	15**/**453,506 (3.31**/**100,000)
Class-3 hospitals	112**/**8,194,585 (1.37/100,000)	128**/**549,267 (23.30/100,000)
Total	126**/**14,109,202 (0.89/100,000)	143**/**1,002,773 (14.26/100,000)

**Figure 3:**
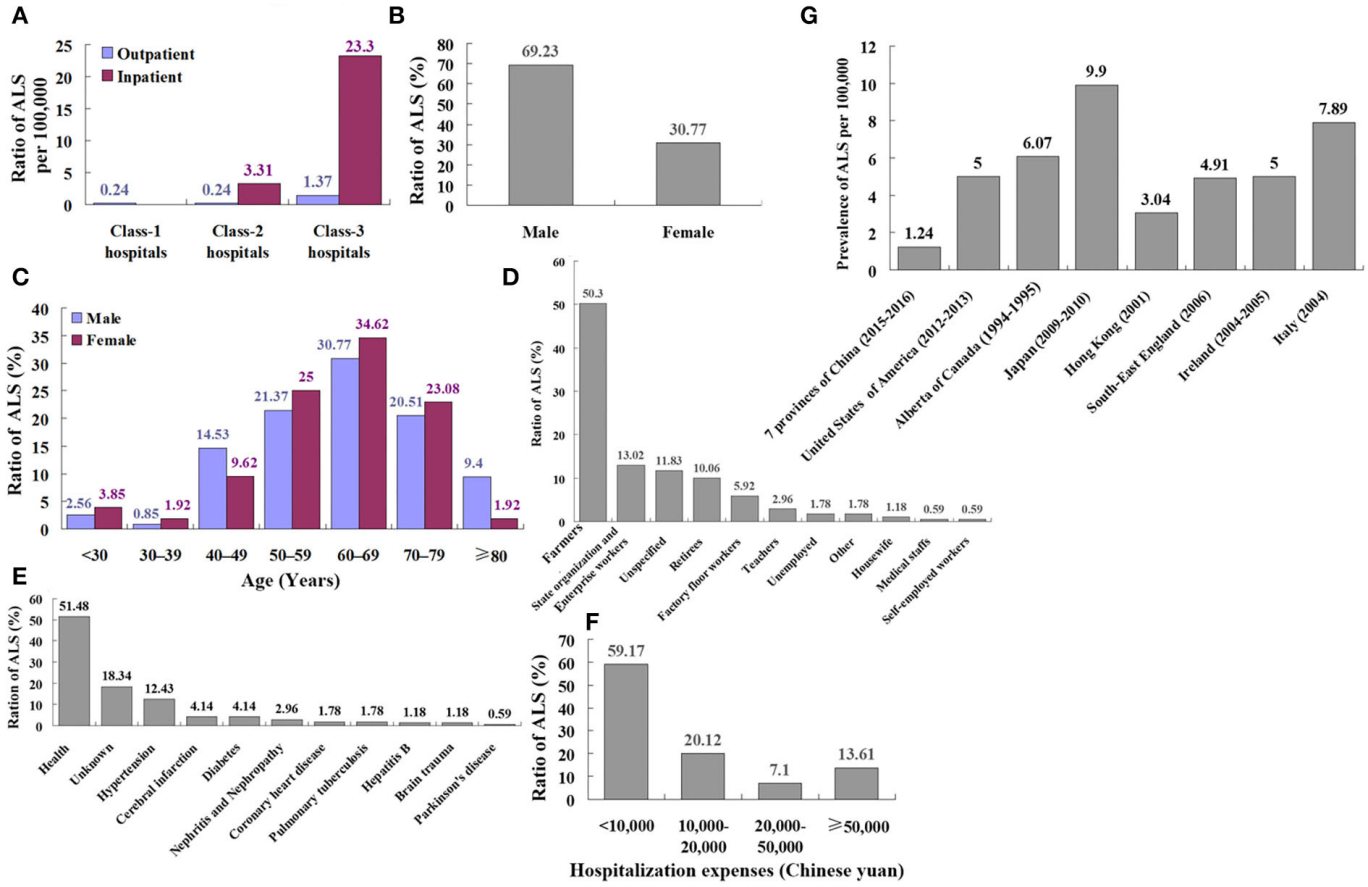
The comparison of survey data based on the hospital. **(A)** The comparison of ALS ratio between outpatients and inpatients in the different level hospitals of China involved 13 class-3 general hospitals, 13 class-2 general hospitals, and 26 street health centers or community health service centers in urban districts, and 13 county-level general hospitals, 39 township health centers and 78 village clinics in rural districts. The prevalence of ALS in inpatients was higher than that in outpatients. The total prevalence of ALS in class-3 hospital patients showed the highest. **(B)** The gender ratio of patients with ALS in the hospital survey, among 169 patients with ALS in the hospital survey, 117 (69.23%) patients with ALS were male and 52 (30.77%) were female. **(C)** The onset age ratio of patients with ALS in the hospital survey, and among those 169 patients, the percentage of 60 to 69 years was the highest, and the percentage of 30 to 39 years was the lowest. **(D)** The profession ratio of patients with ALS in the hospital survey, and among 169 patients with ALS in the hospital survey, farmers were the highest percentage (50.3%), and both medical staffs and self-employed workers were the lowest percentages (0.59%). **(E)** The medical history ratio of patients with ALS in hospital survey, and among 169 patients with ALS in the hospital survey, most patients with ALS had no chronic disease history (51.48%). **(F)** The hospitalization expenses ratio of patients with ALS in the hospital survey, and among 169 patients with ALS in the hospital survey, the hospitalization expenses of most patients with ALS (59.17%) were lower than 10,000 Chinese Yuan. **(G)** The comparison of ALS prevalence in different countries. Our results showed that the prevalence of ALS in seven provinces of the Chinese mainland was 1.24/100,000, which was less than the world other nations and districts including Chinese Hong Kong and similar to the European per year prevalence (2.2/100,000) and United States (1.5/100,000).

All surveyed class-2 hospitals in the hospital survey involved 13 class-2 general hospitals in urban districts and 13 county-level general hospitals in rural districts. There were both outpatient and inpatient departments in these hospitals. Twelve individuals with ALS were identified among 5,082,237 outpatients in these hospitals. Fifteen individuals with ALS were identified among 453,506 inpatients in these hospitals. The prevalence of ALS in class-2 hospital outpatients was 0.24/100,000. The prevalence of ALS in class-2 hospital inpatients was 3.31/100,000 (Table 4, Figure 3A).

Among surveyed 13 class-3 hospitals in urban districts, 102 individuals with ALS were identified among 8,194,585 outpatients in these hospitals And 128 individuals with ALS were identified among 54,9267 inpatients in these hospitals. The prevalence of ALS in class-3 hospital outpatients was 1.37/100,000. The prevalence of ALS in class-3 hospital inpatients was 23.30/100,000 (Table 4, Figure 2A).

One hundred six individuals with ALS were identified among 14,109,202 outpatients in all hospitals. The prevalence of ALS in all hospital outpatients was 0.89/100,000. One hundred forty-three individuals with ALS were identified among 1,002,773 inpatients in all hospitals. The prevalence of ALS in all hospital inpatients was 14.26/100,000. In general, the prevalence of ALS in inpatients was higher than that in outpatients. The total prevalence of ALS in class-3 hospital patients showed the highest (1.37/100,000 outpatients and 23.30/100,000 inpatients) (Table 4, Figure 3A).

Among the 169 patients with ALS in the hospital survey, 117 (69.23%) were men and 52 (30.77%) were women (Figure 3B). The mean age of onset among male patients was 61.43 ± 12.66 years, and the mean age of onset among female patients was 59.98 ± 12.76 years. The percentage of 60 to 69 years was the highest and the percentage of 30 to 39 years was the lowest (Table 5, Figure 3C).

**Table 5:** Epidemiological and clinical characteristics of ALS patients in hospital survey (*n* = 169).

**Characteristics**	**No. cases (n)** ** (Total *n* = 169)**	**Ratio (%)**
Gender		
Male	117	69.23
Female	52	30.77
Age (years)	
Male (Mean age 61.43 ± 12.66)		
<30	3	2.56
30–39	1	0.85
40–49	17	14.53
50–59	25	21.37
60–69	36	30.77
70–79	24	20.51
≥80	11	9.40
Female (Mean age 59.98 ± 12.76)		
<30	2	3.85
30–39	1	1.92
40–49	5	9.62
50–59	13	25.00
60–69	18	34.62
70–79	12	23.08
≥80	1	1.92
Profession	
Farmers	85	50.30
State organization and enterprise workers	22	13.02
Unspecified	20	11.83
Retirees	17	10.06
Factory floor workers	10	5.92
Teachers	5	2.96
Unemployed	3	1.78
Other	3	1.78
Housewife	2	1.18
Medical staffs	1	0.59
Self-employed workers	1	0.59
Medical history	
Health	87	51.48
Unknown	31	18.34
Hypertension	21	12.43
Cerebral infarction	7	4.14
Diabetes	7	4.14
Nephritis and Nephropathy	5	2.96
Coronary heart disease	3	1.78
Pulmonary tuberculosis	3	1.78
Hepatitis B	2	1.18
Brain trauma	2	1.18
Parkinson's disease	1	0.59
Hospitalization expenses ($)	
<10,000	100	59.17
10,000–20,000	34	20.12
20,000–50,000	12	7.10
≥50,000	23	13.61

Among the 169 patients with ALS in the hospital survey, farmers were the highest percentage (50.3%) and medical staffs and self-employed workers were the lowest percentage (0.59%) (Table 2, Figure 3D). Most of the patients with ALS had no chronic disease history (51.48%). About 12.43% of patients had hypertension, 4.14% had cerebral infarction, 4.14% had diabetes, 2.96% had nephritis and nephropathy, 1.78% had coronary heart disease, 1.78% had pulmonary tuberculosis, 1.18% had hepatitis B, 1.18% had brain trauma, and 0.59% had Parkinson's disease (Figure 3E). Hospitalization expenses of most of the patients with ALS (59.17%) were lower than 10,000 Chinese Yuan (Table 5, Figure 3F).

## Discussion

This large, cross-sectional survey study of ALS epidemiology in seven provinces of Chinese eastern, middle, and western regions involved a vast majority of Chinese geographic territory. In this study, we assessed the epidemiological data from population and hospital surveys. Our population survey involved 727,718 individuals including 61.80% (449,718) of Shandong peoples, 22.12% (160,983) of Jiangxi peoples, 7.16% (52,081) of Henan peoples, 2.86% (20,786) of Gansu peoples, 2.60% (18,950) of Sichuan peoples, 2% (14,600) of Jiangsu peoples, and 1.46% (10,600) of Zhejiang people (Table 1).

**Figure 4:**
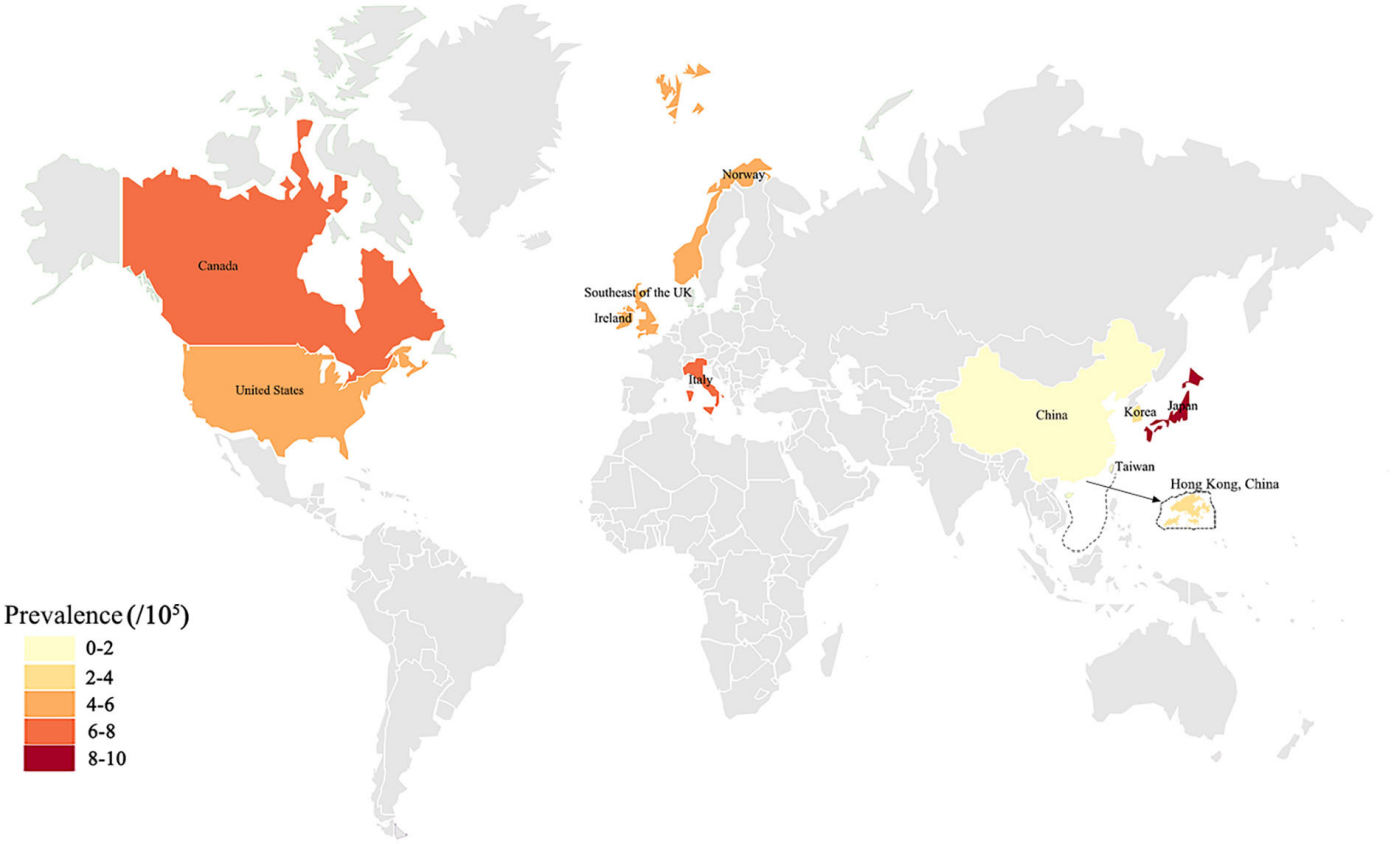
The previously reported ALS prevalence in the global different countries or districts was exhibited on the world map. The deep and shallow color indicated the high and low of ALS prevalence. Deeper color indicated higher ALS prevalence.

**Figure 5:**
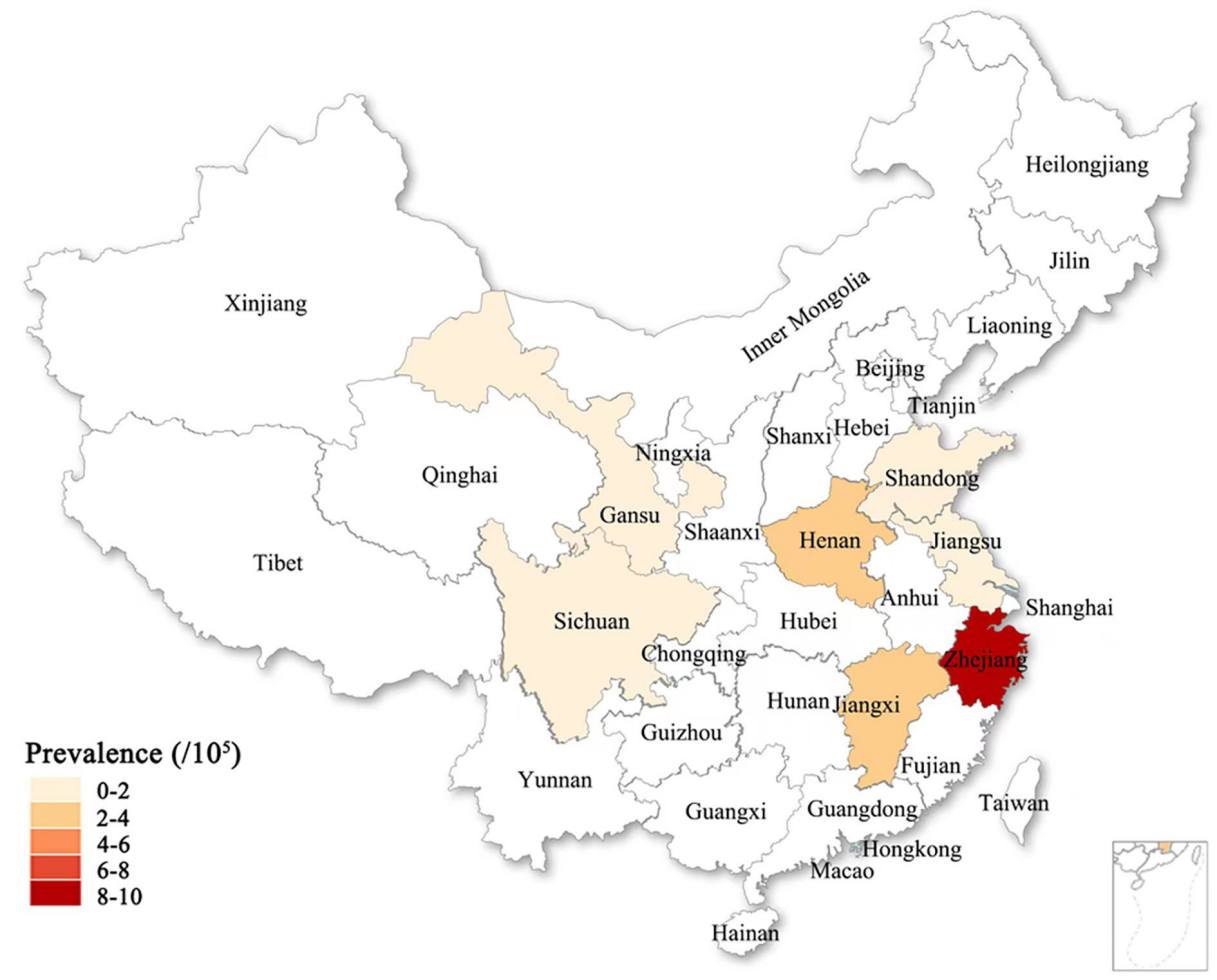
The ALS prevalence in seven provinces of the Chinese mainland in this survey was exhibited on the Chinese map. The deep and shallow color indicated the high and low of ALS prevalence. Deeper color indicated higher ALS prevalence.

Our results showed that the prevalence of ALS in seven provinces of the Chinese mainland was 1.24/100,000, and the total prevalence of ALS was less than that of the world, other nations and districts including Chinese Hong Kong (Table 6, Figures 3G, 4, 5), was similar to the per year prevalence of Europe (2.2/100,000) and United States (1.5/100,000) (Kiernan et al., 2011; Mehta et al., 2016). But in the Chinese mainland, the 9.43/100,000 prevalence of ALS in Zhejiang province was the highest and was adjacent to the world's highest Japan (9.9/100,000) (Table 6, Figures 3G, 4, 5). The secondary was 3.84/100,000 in Henan and 3.73/100,000 in Jiangxi, respectively (Table 1), which was similar to that in the Chinese Hong Kong district (3.04/100,000) (Table 6, Figures 3G, 4, 5). Among them, 65.74% (478,402) were urban people and 34.26% (249,316) were rural people, and it was obvious that the ALS prevalence in rural residents (2.01/100,000) was higher than that in urban residents (0.84/100,000) (Table 2).

**Table 6:** Comparison of ALS/MND prevalence in different countries or districts.

**Countries/Districts**	**Prevalence (per 100,000)**	**Duration**
Seven provinces of China	1.24	2015-2016
United States of America	5.00	2012-2013^[1]^
Alberta of Canada	6.07	1994-1995^[2]^
Japan	9.90	2009-2010^[3]^
Hong Kong	3.04	2001^[4]^
South-East England	4.91	2006^[5]^
Ireland	5.00	2004-2005^[6]^
Italy	7.89	2004^[7]^

^1^Svenson et al., 1999; ^2^Fong et al., 2005; ^3^Abhinav et al., 2007; ^4^Chiò et al., 2009; ^5^Donaghy et al., 2009; ^6^Doi et al., 2014; ^7^Mehta et al., 2016.

Of the nine definite ALS in the population survey, 77.78% were farmers, occupying more than 50% among them (Table 3). About 22.22% had an ALS family history, which was slightly more than the international reported ~10% (Table 3), and 77.78% were sALS, which was slightly less than the international reported approximate 80% (Table 3) (Zarei et al., 2015). The survival duration of sALS was 0.5–4 years and the average survival was 2.07 ± 1.08 years, which was less than the previously reported around 3.5 years (Table 3) (Salameh et al., 2015). The survival duration of fALS ranged from 6 to 8 years in this survey. fALS in the previous survey existed a longer survival duration, someone lives more than 10 years, even exceeding 30 years or more long (Salameh et al., 2015). About 77.78% were male patients, 22.22% were female patients, male patients were significantly higher than female patients (Table 3), which was consistent with the information reported by previous investigation (Kiernan et al., 2011).

In the hospital survey, the prevalence of ALS was 0.24/100,000 in 832,380 outpatients in class-1 hospitals (Table 4). The prevalence of ALS was 0.24/100,000 in 5,082,237 outpatients and 3.31/100,000 in 453,506 inpatients in class-2 hospitals, respectively (Table 4, Figure 3A). The prevalence of ALS was 1.37/100,000 in 8,194,585 outpatients and 23.30/100,000 in 54,9267 inpatients in class-3 hospitals, respectively (Table 4, Figure 3A). The prevalence of ALS was 0.89/100,000 in 14,109,202 outpatients and 14.26/100,000 in 1,002,773 inpatients in all hospitals, respectively. There could be no doubt that the total prevalence of ALS in class-3 hospitals was the highest and the secondary was in class-2 hospitals. It was affirmative that the prevalence of ALS in both inpatients and outpatients in the class-3 hospital was higher than that in both class-2 and class-1 hospitals (Table 4, Figure 3A). The possible cause of the highest prevalence in class-3 hospitals is that ALS is a rare disease and the patients with ALS themselves are not completely confident of the diagnosis made by doctors in both class-1 and class-2 hospitals, as well as the doctors in both class-2 and class-1 find it difficult to diagnose ALS because of the limitation of doctor diagnostic ability and hospital medical condition.

Among the 169 definite patients with ALS in the hospital survey, 69.23% were male and 30.77% were female (Figure 3B), which was similar to the previous investigation reported information (Kiernan et al., 2011). The male mean age of onset was 61.43±12.66 years, the female mean age of onset was 59.98 ± 12.76 years, the peak onset age was at 60 to 69 years, and the lowest age of onset was 30 to 39 years (Table 5, Figure 3C). Among different professions, 50.3% were farmers, and medical staffs and self-employed workers were the lowest, only occupying 0.59% (Table 2, Figure 3D). About 51.48% of ALS had no chronic disease history (Figure 2E). The hospitalization expenses of 59.17% of patients with ALS were lower than 10,000 Chinese Yuan (Table 5, Figure 3F).

We conducted informative comparisons with previous studies in this study. Although the prevalence, onset age, geographical location, profession, chronic disease history, familial history, survival duration, and diagnostic and cured condition (Hospitalization expenses) of ALS in our study were slightly different than the corresponding rates in the previous study, the overall results were similar to the previous investigation data (Kiernan et al., 2011; Salameh et al., 2015). We speculate that the relatively low prevalence of ALS observed in our study implies greater omit diagnosis in the Chinese mainland because the criteria of ALS cannot be accurately realized by doctors in under class-2 hospitals. Although both population and hospital survey studies showed an increased risk of farmers and rural residents, our study did not show a statistically significant association between farmers, rural residents, and the development of ALS because of the limited sample. The multiple coexisting conditions seen in farmers and rural residents may confer greater susceptibility to ALS, such as associated environments, education and physical power, and so on, which is, in part, supported by the higher prevalence seen in our study than in previous studies (Ludolph et al., 2012; Zarei et al., 2015; Vasta et al., 2022). The profession in agriculture was risk work for the development of ALS in the Chinese mainland. As far as know, the information that the higher prevalence of ALS in rural residents and farmers was not reported before our study.

Our study demonstrated that the prevalence of ALS and hospitalization expenses were lower than global other countries and districts. For that, we doubted that the survey results did not represent that the prevalence of ALS in our country was really lower than in other countries and districts in the world. In fact, it might be that we weren't enough know the disease of ALS, especially for the non-neurological doctor, omitted lots of patients with ALS. Moreover, the major reason for lower hospitalization expenses might not be because patients with ALS do not have enough money for hospitalization cure but the ALS treatment has exerted the national medical insurance of chronic disease in our country, and patients with ALS do not need to pay too much money for hospitalized treatment and the fact that mainly because lots of non-neurologists did not generally and comprehensively understand the normal processes of ALS diagnosis and treatments, which resulted in inaccurate diagnosis and treatment of ALS. This finding questions in our study reminder us to nationwide efforts to prevent to omit the diagnosis and the hospitalized treatment of ALS by spreading knowledge about the ALS diagnosis and treatment in popular peoples and non-neurological doctors, enhancing the training of ALS diagnosis and treatment in neurological doctors, improving the ratio of ALS diagnosis and the hospitalized treatment, and reducing the ratio of omitted diagnosis, providing better medical service for patients with ALS and improving the life quality of patients with ALS.

Our data further revealed that men were more likely to suffer from ALS than women. The survival duration of sALS was about 3 years and the survival duration of fALS was longer than that of sALS (Kiernan et al., 2011; Salameh et al., 2015). In addition, it seems that the peak onset age (60–69 years) in our study is later than the previous investigation data (Kiernan et al., 2011), for that, we thought that it could not completely exclude some patients with ALS or diagnosed by doctors at the initial or early stage of ALS, and it also could not completely exclude the retrospective survey of disease resulted in the mistaken judgment of accurate onset time.

Our study has some strengths. Our survey involved the representative regions of the population and hospitals in the Chinese mainland, which enabled the general evaluation of prevalence, sexuality, onset age, profession, survival time, and diagnostic and cured condition of ALS in the Chinese mainland. Of course, our study also exist some limitations, such as our study did not include all population and hospitals in the Chinese mainland, and also the survey of multiple centers. Therefore, our study might have some deficiencies and errors.

## Conclusion

In conclusion, the ALS hospitalization expenses in our survey were the lowest compared with the previously reported countries and districts. The farmer was a possible higher risk profession for ALS, and the majority of ALS were not accompanied by other chronic diseases. Besides, the general epidemiology features in our study were not found to be significantly different from the previous epidemiology survey. The prevalence, sexuality, onset age, and survival time of ALS were similar to the reported information in the previous epidemiological studies conducted in other countries and districts (Svenson et al., 1999; Fong et al., 2005; Abhinav et al., 2007; Chiò et al., 2009; Donaghy et al., 2009; Kiernan et al., 2011; Doi et al., 2014; Salameh et al., 2015; Mehta et al., 2016).

## Data availability statement

The original contributions presented in the study are included in the article/supplementary material, further inquiries can be directed to the corresponding authors.

## Ethics statement

We declare that all human studies have been approved this survey by the Ethics Committee of First Affiliated Hospital of Nanchang University (Approval number: 2014-06-01). All participants were invited to participate voluntarily. The study protocol and procedures follow the ethical guidelines of the 1975 Declaration of Helsinki. A verbal informed consent was obtained from all subjects in this study in approved by from the local Center for Disease Control, selected hospitals, and selected communities and villages, which gave approval for verbal consent. Written informed consent for participation was not required for this study in accordance with the national legislation and the institutional requirements.

## Author contributions

JZ, XL, HL, SX, XW, and RX conceived the idea, designed the research, and wrote the manuscript. JZ, XL, HL, and SX performed the data collection, extraction, and analyses. XW and RX contributed to the data verification. All authors read, reviewed, and approved the final manuscript.

## Funding

This work was supported by the funding from the National Natural Science Foundation of China (30560042, 81160161, 81360198, and 82160255), Education Department of Jiangxi Province (GJJ170042, GJJ13198, and GJJ170021), Jiangxi Provincial Department of Science and Technology ([2014]-47, 20142BBG70062, 20171BAB215022, and 20192BAB205043), Health and Family Planning Commission of Jiangxi province (20181019), and Jiangxi Provincial Department of Science and Technology Gan Po Elite 555 (Jiangxi Finance Elite Education Refers to [2015] 108). The funding sources were not involved in the study design, data collection, data analyses, interpretation, or writing of this manuscript.

## Conflict of interest

The authors declare that the research was conducted in the absence of any commercial or financial relationships that could be construed as a potential conflict of interest.

## Publisher's note

All claims expressed in this article are solely those of the authors and do not necessarily represent those of their affiliated organizations, or those of the publisher, the editors and the reviewers. Any product that may be evaluated in this article, or claim that may be made by its manufacturer, is not guaranteed or endorsed by the publisher.

## Author disclaimer

The content is solely the responsibility of the authors and does not necessarily represent the official views of the grant foundation.
